# A Case of Diptheria

**Published:** 1895-10

**Authors:** S. Mills Fowler

**Affiliations:** Chicago, Ill.


### A CASE OF DIPHTHERIA.

S. Mills Fowler, M. D., Chicago, Ill.

Master K., aged three years.

When I first saw this little patient he had been sick for three days, and the parents becoming alarmed, thought it advisable to call in a medical man to share the responsibility of his death.

I found him lying on his side with his mouth partly open, with a heavy sort of snoring respiration and a bloody, excoriating watery fluid running from his nose, and the characteristic odor of diphtheria in the room.

A dirty, grayish white coating covered nearly the whole of the pharynx and fauces, being thickest on the left side. The tonsils and pharynx were much swollen and of a dark purplish red. The neck and throat were swollen on the outside, and very sensitive to touch. Although he seemed in a decided stupor, he did not want anything to come in contact with these parts, and would involuntarily pull at the clothing around the throat. He did not want to be handled or touched; it seemed to cause pain; but preferred to be left quietly on his bed.

Lachesis, which was the only remedy that came to my mind, seemed only to palliate; it arrested the progress of the disease, and that was all. It was tried first in the CM, and later in both higher and lower potencies. The child did not improve, but it got no worse.

After two days of this sort of waiting anxiety, and not being able to settle upon any other remedy, I gave one dose of Diph.$^{cmm}$, Swan, dry on the tongue, which acted charmingly, and the little fellow was convalescent in two days.

